# Antimicrobial and Probiotic Properties of Yeasts: From Fundamental to Novel Applications

**DOI:** 10.3389/fmicb.2012.00421

**Published:** 2012-12-19

**Authors:** Rima Hatoum, Steve Labrie, Ismail Fliss

**Affiliations:** ^1^Nutraceuticals and Functional Foods Institute, STELA Dairy Research Centre, Université LavalQuébec, QC, Canada

**Keywords:** yeasts, antagonistic activities, probiotic, killer toxin, mycocin, veterinary, medical

## Abstract

The yeasts constitute a large and heterogeneous group of microorganisms that are currently attracting increased attention from scientists and industry. Numerous and diverse biological activities make them promising candidates for a wide range of applications not limited to the food sector. In addition to their major contribution to flavor development in fermented foods, their antagonistic activities toward undesirable bacteria, and fungi are now widely known. These activities are associated with their competitiveness for nutrients, acidification of their growth medium, their tolerance of high concentrations of ethanol, and release of antimicrobial compounds such as antifungal killer toxins or “mycocins” and antibacterial compounds. While the design of foods containing probiotics (microorganisms that confer health benefits) has focused primarily on *Lactobacillus* and *Bifidobacterium*, the yeast *Saccharomyces cerevisiae* var. *boulardii* has long been known effective for treating gastroenteritis. In this review, the antimicrobial activities of yeasts are examined. Mechanisms underlying this antagonistic activity as well as recent applications of these biologically active yeasts in both the medical and veterinary sectors are described.

## Introduction

The term “yeast” was derived originally from the Dutch word “*gist*,” which refers to the foam formed during the fermentation of beer wort. Other words referring to yeast, such as the French word “*levure*,” refer to the role of yeast in causing bread dough to rise. Few other microbial organisms match the yeasts in terms of historical, economic, and scientific significance. In addition to their role in the production of fermented foods and beverages, the yeasts play various roles in livestock feeding and veterinary practices as well as in medicine and the biomedical and pharmaceutical industries. One of these roles consists primarily of antagonizing other microorganisms such as undesirable yeasts, molds, and bacteria.

The inhibitory activity of yeast was discovered first by Hayduck (1909). Somewhat later, other researchers reported the antagonistic action of yeasts against other yeasts, involving the production of secondary metabolites known as killer toxins or “mycocins” (Young and Yagiu, 1978; Rosini and Cantini, 1987; Walker et al., 1995; Suzuki et al., 2001; Marquina et al., 2002). In summarizing the antimicrobial effects of yeasts present in fermented foods and beverages, Viljoen (2006) mentions actions of organic acids, antibiotic factors, volatile acids, hydrogen peroxide, and various other substrates excreted in the product. However, there has been relatively little study devoted to identifying the mechanisms of inhibition by yeasts.

In this chapter, the metabolic factors mentioned above as well as recent advances in knowledge of the antagonistic properties of yeasts will be discussed, with emphasis on the potential opportunities for application in various fields. Although only *Saccharomyces cerevisiae* var. *boulardii* has been studied in detail and its inhibitory mechanisms well defined, significant antagonistic activities have been associated with several other genera or species, and exploration of their potential industrial and biotechnological applications is expected.

## Yeasts

### General consideration and taxonomy

The yeasts described in this review are all members of the phylum *Ascomycota* and the class *Saccharomycota*. Phylogenetic analysis of the phylum *Ascomycota* has significantly changed yeast classification in recent years (Hibbett et al., 2007; Kurtzman et al., 2011a,b). Yeasts are eukaryotic microorganisms widespread in natural environments including the normal microbial flora of humans, on plants, on airborne particles, in water, in food products, and in many other ecological niches. Yeasts are important in many complex ecosystems, as frequent early colonizers of nutrient-rich substrates (Kurtzman et al., 2011a,b). They are involved in many interactions with other microorganisms, including symbiosis, mutualism, parasitism, and competition. They also exhibit both asexual and sexual states. The asexual state of given yeast is called the anamorph, while the sexual state is the teleomorph. One result of this phenomenon is that there is a valid Latin name for each state, since no teleomorph has been found for many asexual forms or because the phylogenetic relationship between anamorph and teleomorph has not been confirmed.

The most common mode of vegetative growth of yeasts is by budding, which may be blastic or thallic. Anamorphic and teleomorphic genera may grow either as a “yeast-like” unicellular organism or as a “mold-like” filamentous organism, a phenomenon called dimorphism. Moreover, some species are able to form a true mycelium, while genera such as *Candida* produce a well-developed pseudomycelium, or both pseudo and true mycelium in the case of *Candida tropicalis* (Goldman, 2008).

Among the yeasts belonging to the phylum *Ascomycota*, the genus *Saccharomyces* is the most studied. Many of the approximately 20 species of this genus are of great biotechnological significance due to applications including alcoholic fermentation, bread-making, single cell protein, vitamin production, synthesis of recombinant proteins, and biological control (Webster and Weber, 2007). The most significant species is certainly *S. cerevisiae* (baker’s and brewer’s yeast), due to its economic impact. *S. cerevisiae* is used for the annual production of an estimated 60 million tons of beer, 30 million tons of wine, 800,000 tons of single cell protein, and 600,000 tons of baker’s yeast (Pretorius et al., 2003). The vegetative cells of *S. cerevisiae* are normally diploid, but some strains have been reported as aneuploid or tetraploid (Webster and Weber, 2007). Over the past four decades, a yeast first identified as *Saccharomyces boulardii* has been studied for its potential probiotic use (Buts, 2009). The taxonomic position of *S. boulardii* was determined using multi locus sequence analysis targeting the D1/D2 domain of the 26S rDNA subunit, the ITS1-5.8S rDNA-ITS2 sequence, and the mitochondrial cytochrome C oxidase II (COX2) gene. Each locus is highly similar to the corresponding loci in *S. cerevisiae*, which led to the proposal to assimilate *S. boulardii* into the *S. cerevisiae* species (Van Der Aa Kühle and Jespersen, 2003). The denomination *S. cerevisiae* var. *boulardii* has been proposed, but the designation *S. boulardii* is still largely used in the scientific literature.

### Yeast metabolism

Yeasts are heterotrophic organisms, meaning that energy metabolism and carbon metabolism are intimately interconnected. Adenosine triphosphate (ATP) is provided by oxidation of organic molecules that also act as carbon sources for biosynthesis, and is ultimately used as the energetic intermediate for practically all cellular activities (Rodrigues et al., 2006). Yeasts have relatively simple nutritional requirements, a carbon source, a nitrogen source (ammonium salt, nitrate, amino acids, peptides, urea, purines, pyrimidines), phosphate, sulfate, lower concentrations of potassium, magnesium, calcium, iron, zinc, and in most cases a vitamin such as biotin, thiamine, or pantothenic acid making up a complete growth medium. It is well known that the principal carbon source employed by yeasts is carbohydrate, primarily hexose sugars as monosaccharides (glucose, fructose, galactose, or mannose) or disaccharides (maltose or sucrose). In addition, a wide range of other carbon sources (e.g., alcohols, organic acids) can be utilized under aerobic conditions (Deak, 2006).

Van Dijken and Scheffers (1986) classified yeasts physiologically according to the type of energy-generating process involved in sugar metabolism, namely non-fermentative, facultatively fermentative, or obligately fermentative. It was later found that basidiomycetous yeasts such as *Cryptococcus*, *Rhodotorula*, and others are non-fermentative and strictly aerobic (Goldman, 2008). Not even the obligate fermentative species can survive for very long under strict anaerobic conditions, since the synthesis of certain membrane constituents (i.e., sterols) requires oxygen (Deak, 2006). Yeast metabolism and physiology are thus strongly dependent on sugar and oxygen availability. Yeast aerobic respiration has been defined by Dawes (1986) as the complete oxidation of carbon-containing molecules to CO_2_ and H_2_O by the interrelated processes of the tricarboxylic acid (TCA) cycle and the electron transport chain coupled to phosphorylation with oxygen as the terminal electron acceptor. In yeast anaerobic metabolism, often called “alcoholic fermentation,” pyruvate produced by glycolysis is split into ethanol and CO_2_ in a redox-neutral process (Van Dijken and Scheffers, 1986).

Finally, there are three frequently observed effects associated with the type of energy-generating processes involved in sugar metabolism and/or oxygen availability; Pasteur, Cabtree, and Custer effect (Van Dijken and Scheffers, 1986; Fredlund et al., 2004; Dickinson and Kruckeberg, 2006; Wijsman et al., 1984). Due to its industrial importance, better understanding of yeast metabolism is needed in order to provide insight into the formation of primary and secondary metabolites and their impact on human health.

## Antagonistic Characteristics of Yeasts

The use of antagonistic bacteria to inhibit pathogenic bacteria has been studied extensively over the years, while little attention has been given to yeasts in a similar role. The study and potential applications of antibacterial compounds secreted by yeasts are therefore still at an early stage of development.

**Figure 1:**
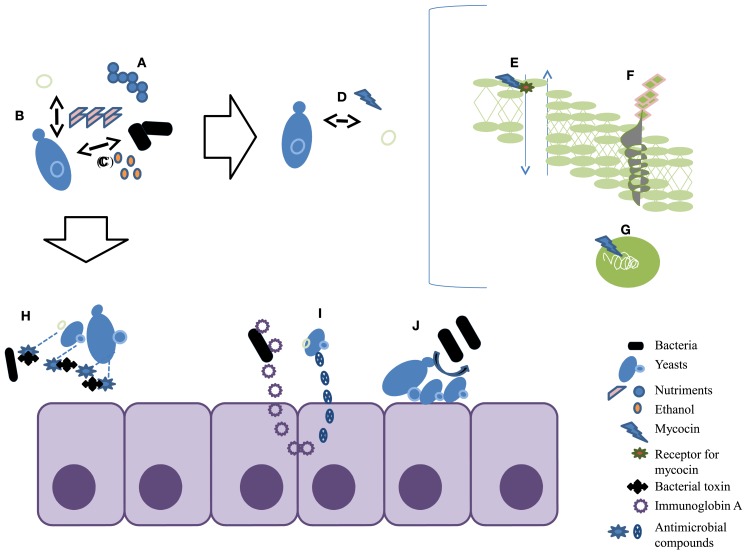
**Summary of the different aspects of antagonistic properties of yeasts**. **(A)** Competition for nutrients; **(B)** pH changes; **(C)** production of high concentrations of ethanol; **(D)** killer toxins or mycocins; **(E)** mycocin causes ion leakage by the formation of channels on the cytoplasmic membrane; **(F)** mycocin inhibits the synthesis of cell wall component β-1,3-glucan; **(G)** mycocin interrupts cell division by blocking the DNA synthesis; **(H)** proteases degrade bacterial toxins; **(I)** stimulate the immune response; **(J)** yeasts inhibit attachment to intestinal cells.

Antagonism of microorganisms by yeasts has been attributed primarily to (1) competition for nutrients, (2) pH changes in the medium as a result of growth-coupled ion exchange or organic acid production, (3) production of high concentrations of ethanol, (4) secretion of antibacterial compounds and release of antimicrobial compounds such as killer toxins or “mycocins” (Suzuki et al., 2001; Golubev, 2006; Young and Yagiu, 1978). Mycocins are extracellular proteins or glycoproteins that disrupt cell membrane function in susceptible yeasts, which bear receptors for the compound (Golubev, 2006). Their activity is directed primarily against yeasts closely related to the producer strain, which has a protective factor. The first mycocins were identified in association with *S. cerevisiae* in the brewing industry (Bevan and Somers, 1969). Several have since been isolated, frequently where yeast populations exist in high density and in highly competitive conditions. Mycocin production occurs among many yeast genera including *Saccharomyces*, *Candida*, *Cryptococcus*, *Debaryomyces*, *Kluyveromyces*, *Pichia*, *Torulopsis*, *Williopsis*, and *Zygosaccharomyces* (Young and Yagiu, 1978; Magliani et al., 1997; Chen et al., 2000; Schmitt and Breinig, 2002; Golubev, 2006; Hodgson et al., 1995). Genetic and molecular studies have shown that the killer toxin trait may be carried on extra-chromosomal elements in the form of double-stranded RNA viruses (Wickner, 1996), on double-stranded linear DNA (Gunge et al., 1981; Hayman and Bolen, 1991), or on a chromosome (Kimura et al., 1993; Suzuki and Shimma, 1999). The well known mechanisms of the killer toxin are the interruption of cell division by blocking the DNA synthesis, inhibition of synthesis of the cell wall component β-1,3-glucan (Izgu and Altinbay, 2004), and ion leakage caused by the formation of channels on the cytoplasmic membrane (Kagan, 1983; Schmitt and Breinig, 2002; White et al., 1989; Figure 1). Unlike yeast-against-yeast antagonism, the antibacterial properties of yeast are much less documented.

**Figure 2:**
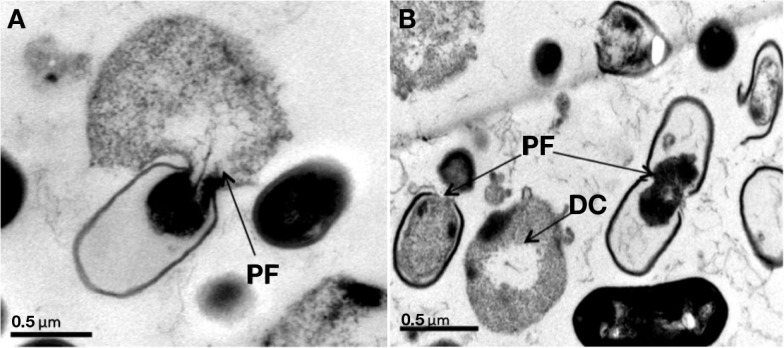
**(A,B)** TEM micrographs showing *Listeria monocytogenes* LMA-1045 after 1 min of contact with solvent extracts of Wickerhamomyces *anomalus* LMA-827 **(A)** and *Candida tropicalis* LMA-693 **(B)** culture supernatants. PF – pore formation in cell membrane; DC – digested cell. Grids were examined at 80 kV. The magnification factor is 30,000×. Bars indicate 0.5 μm.

Historically, the first positive indications of the antagonistic activity of yeasts published early in the twentieth century by Hayduck (1909) and Fernbach (1909; cited in Golubev, 2006) who reported a volatile thermolabile toxic extract from yeast probably an amine that inhibits the growth of *Escherichia coli* and Staphylococci (Viljoen, 2006). Fatichenti et al. (1983) showed that the antibacterial activity of *Debaryomyces hansenii* against *Clostridium tyrobutyricum* and *Clostridium butyricum* was related to its ability to produce both extracellular and intracellular antimicrobial compounds. Bilinski and Casey (1989) reported inhibition of the growth of the beer spoilage bacteria *Bacillus megaterium* and *Lactobacillus plantarum* due to the conversion of methylene blue into a pharmacologically active form by *Kloeckera apiculata* and *Kluyveromyces thermotolerans*. Dieuleveux et al. (1998) subsequently described inhibition of *Listeria* by a strain of *Geotrichum candidum* isolated from French red smear cheese. The two anti-listerial compounds (d-3-phenyllactic and d-3-indollactic acids) are stable over a wide pH range and can be heated to 120°C for 20 min. Also, Cavalero and Cooper (2003) demonstrated that *Candida bombicola* produces extracellular glycolipids called sophorosides, which have proven antibacterial activity against *Staphylococcus aureus* and also inhibit *Candida albicans*. Having tested hundreds of dairy yeasts, Goerges et al. (2006) reported a strain of *Candida intermedia* capable of reducing viable *Listeria* counts by 4 log CFU/cm^2^ in co-culture on agar, while three *C. intermedia* and one *Kluyveromyces marxianus* suppressed *L. monocytogene*s growth by 3 log CFU/cm^2^. The same group more recently found a strain of *Pichia norvegensis* (WSYC 592) able to reduce *L. monocytogenes* counts by 7 log cycles, while numerous strains of *Issatchenkia orientalis*, *Candida krusei*, and *K. marxianus* reduced *Listeria* counts by 4–5 log units in co-culture on agar (Goerges et al., 2011). However, strain WSYC 592 decreased *Listeria* counts on Tilsit cheese by only one log cycle. More recently, Hatoum et al. (2012) characterized anti-listerial hydrophobic peptides extracted from cultures of four wild dairy yeasts identified as *D. hansenii*, *P. fermentans*, *C. tropicalis*, and *W. anomala*. In experiments using a Camembert curd model, the anti-listerial compounds of *D. hansenii* and *W. anomalus* were found to reduce *L. monocytogenes* counts by 3 log units during the first 9 days of ripening. The active principles are thermostable and apparently peptides and appear to induce leakage in bacterial cells and ultimately cause bacterial lysis (Figure 2).

## Applications of Antagonistic Activities of Yeasts

The discovery of antagonistic activities of yeasts has had a significant impact in numerous fields such as food, agriculture, medicine, veterinary medicine, environmental protection, and others. The following sections of this paper provide an updated summary of published findings regarding the antagonistic properties of yeasts. **Figure 3** presents an organogram in which the applications are grouped in three different clouds in a free-mind program http://bactibase.pfba-lab-tun.org/freemind/. The articles are linked to these three clouds.

### Food and agriculture

#### Processed food and beverages applications

The past decades have witnessed the application of antagonistic yeast starter cultures in various food processing industries. It is well known that final product quality in industries such as wine-making, sausage production, cheese ripening, bakery, and the “fermentations” of cacao and coffee beans is affected directly by the development of spoilage microorganisms (Romano et al., 2006; Viljoen, 2006). Antagonistic yeasts starter cultures contribute to product safety primarily by inhibiting pathogen growth during fermentation, and to finish product sensory qualities and shelf-life by inhibiting spoilage organisms.

Numerous studies have proposed the use of mycocin-producing yeasts as starter cultures to prevent the growth of spoilage yeast strains and secondary fermentation wines (Hara et al., 1980; Pfeiffer and Radler, 1984; Boone et al., 1990; Van Vuuren and Jacobs, 1992; Comitini et al., 2004a,b; Seki et al., 1985). A wine starter culture such as *S. cerevisiae* is normally able to dominate native yeasts in the grape must during fermentation (Pretorius, 2000). Also, in the production of sparkling wine, Todd et al. (2000) studied the behavior of two sensitive strains of *S. cerevisiae* in the presence of a mixture of two K2 killer toxin. The authors concluded that this interaction accelerates the yeast autolysis and per consequence the release of proteins that affects the end product quality. Although *S. cerevisiae* strains that produce K2 toxins have been found effective for preventing the growth of spoilage yeast strains (Pfeiffer and Radler, 1984; Van Vuuren and Jacobs, 1992), numerous non-*Saccharomyces* yeasts present on the surface of grapes are insensitive to the *S. cerevisiae* killer toxins. Killer toxins secreted by *Kluyveromyces phaffii* (KpKt) are strong inhibitors of *Hanseniaspora uvarum* (Ciani and Fatichenti, 2001; Michalcáková et al., 1993) and thus appeared to have bio-preservative properties potentially useful for the wine industry. Years later, Comitini et al. (2004a,b) purified and characterized a glycosylated 33 KDa protein produced by *K. phaffii* and found that it binds to β-1,3-glucan, causing the formation of pores in the cell walls of wine spoilage yeasts. They also showed that killer toxins secreted by *W. anomalus* and *Kluyveromyces wickeramii* inhibit *Dekkera* and *Brettanomyces*, spoilage yeasts that cause unpleasant odors in wine. Numerous studies have confirmed remarkable inhibitory properties of the killer toxin NCYC 432 (49 kDa glycosylated peptide) of *W. anomalus* and the mycosin HMK of *Williopsis markii* (Ibeas et al., 1996; Izgu and Altinbay, 2004; Izgu et al., 2006; Lowes et al., 2000) against a wide range of pathogenic and spoilage fungi. The antagonistic properties of yeast can also influence the interactions of wine yeast and malolactic bacteria mainly *Oenococcus oeni*. In particular, this interaction between yeast and bacteria can stimulate or prevent the progress of malolactic fermentation which improves wine stability and quality (Alexandre et al., 2004).

Applications of the mycocin-producing yeasts have been suggested also for olive fermentation (Llorente et al., 1997; Asehraou et al., 2007; Hernández et al., 2008; Marquina et al., 1992); beer production (Young, 1981) sake production (Yoshiuchi et al., 2000), and in salted fermented foods such as miso, soy sauce, and salted vegetables (Kono and Himeno, 1997; Suzuki et al., 2001). It has been reported that the inhibitory activity of *D. hansenii* is enhanced by the presence of NaCl (Llorente et al., 1997), which enhance the cell membrane porosity. This phenomena has also been reported in dough production (Almeida and Pais, 1996) and bread (Bortol et al., 1986). Uses of mycocin-producing starter yeasts to prevent spoilage in yogurt (Lowes et al., 2000; Liu and Tsao, 2009b,c, 2010a,b), probiotic cheese (Jakobsen and Narvhus, 1996), and other foods (Palpacelli et al., 1991) have also received attention.

In spite of their potential benefits in terms of food preservation, mycocin-producing yeasts should be used with caution. The strain might have a negative impact on end product quality, as has been observed for cheese (Valdés-Stauber et al., 1997; Wyder and Puhan, 1999) and wine (Fleet, 2007). Some strains have developed super killer toxins and their escape into the natural environment could threaten other industrial fermentation processes (Magliani et al., 2005). Given the wide range of biotechnological applications of antagonistic yeasts, further studies are required to standardize inoculation parameters and maximize end product stability.

#### Bio-control in unprocessed foods

The antagonistic properties of yeasts have been used in numerous promising agricultural applications as natural bio-control agents, both as soil treatments and for preventing diseases in pre- and post-harvest crops. In 1995, the USA environmental protection agency registered *Candida oleophila* as bio-control post-harvest yeast (El-neshawy and Wilson, 1997). This application has been discussed in numerous studies (Walker et al., 1995; Lowes et al., 2000; Masih et al., 2001; Masih and Paul, 2002; Santos and Marquina, 2004; Spadaro and Gullino, 2004; Kitamoto et al., 1999).

### Medical applications

#### Probiotics

One of the most significant non-process uses of yeasts is as probiotic microorganisms. The Food and Agriculture Organization (FAO) of the World Health Organization (WHO) defines probiotics as “live microorganisms which when administered in adequate amounts confer a health benefit on the host” (FAO/WHO, 2002). For many years, probiotics were used only in animal feeds. Various authors have shown that surplus biomass from the fermentation industry, recycled as an additive to cattle, hog, and poultry diets, improves livestock performance and product quality (Dawson et al., 1990; Li et al., 2006; van Heugten et al., 2003). It was hypothesized that the yeast *S. cerevisae* provides vitamins B and organic acids that stimulate the rumen acetogens (Chiquette, 2009) which form acetic acid (a utilizable form of carbon) from hydrogen and carbon dioxide.

First isolated from litchi fruit in Indochina and described in 1984 as a saprophytic yeast, *S. cerevisiae* var. *boulardii* has emerged as a probiotic species for human consumption (Van Der Aa Kühle et al., 2005). This strain has also been recommended for the prevention and treatment of several types of gastroenteritis in children and adults (Kurugöl and Koturoglu, 2005; Htwe et al., 2008). Nowadays, probiotic yeasts can be delivered either in fermented foods or as lyophilized cultures administered orally, for example, to patients who have been hospitalized as a consequence of severe diarrhea. Several yeast species, including *D. hansenii*, *Torulaspora delbrueckii* (Psani and Kotzekidou, 2006), *Kluyveromyces lactis*, *Yarrowia lipolytica* (Chen et al., 2010), *K. marxianus*, *K. lodderae* (Kumura et al., 2004) have been found strongly antagonistic to pathogenic bacteria and to tolerate passage through the gastrointestinal tract. In a recent *in vitro* study, Etienne-Mesmin et al. (2011) investigated the probiotic effect of *S. cerevisiae* CNCM I-3856 against *E. coli* O157:H7. The results showed that the probiotic yeast exert the antagonistic effects in the distal part of the small intestine and that might be due to ethanol production. However, only *S. boulardii* is considered as a probiotic (Czerucka et al., 2007). Its probiotic efficiency has been well documented in numerous clinical studies (Sazawal et al., 2006).

The text that follows describes the various mechanisms that underlie the probiotic activities of *S. boulardii* against a variety of pathogens such as *Escherichia coli*, *Vibrio cholera*, *Clostridium difficile*, and *Samonella* in clinical and animal studies.

##### Antibiotic associated diarrhea “AAD”

Antibiotic therapy is well known to destroy the normal bacterial population of the digestive tract, which allows harmful bacteria to colonize and irritate the host gut and cause antibiotic associated diarrhea (Coté and Buchman, 2006). Numerous placebo-controlled clinical studies have shown the beneficial effects of *S. boulardii* in preventing antibiotic associated diarrhea (D’Souza et al., 2002; Erdeve et al., 2004; Duman et al., 2005; Kotowska et al., 2005; Can et al., 2006; Cindoruk et al., 2007; Whelan, 2007; Bravo et al., 2008; Lewis et al., 1998). In a double-blind study, Adam et al. (1976) reported a significant reduction in AAD symptoms in the group that received 200 mg of *S. boulardii* (10^9^ CFU/day) for 7 days. Only 4.5% of this group developed AAD, compared to 17.5% of the placebo group. In a study involving 193 hospitalized patients receiving β-lactam antibiotics (Mcfarland et al., 1994), one group received 1 g of *S. boulardii* while the other was given a placebo, both for periods lasting 3 days beyond the antibiotic treatment. AAD appeared in 7.2% of the *S. boulardii* group compared to 14.6% (*P* < 0.05) of the placebo group. In a study of the effect of 200 mg of *S. boulardii* (10^9^ CFU/day) for 2 weeks beyond the antibiotic treatment, Surawicz et al. (1990) reported AAD in 9.5% of the probiotic-treated group versus 21.8% (*P* < 0.05) in the placebo group. In a study (involving 151 patients) of the preventive effect of *S. boulardii* given only for the duration of the antibiotic treatment, Can et al. (2006) found AAD in 1.4% of the probiotic-treated group versus 9.0% in the placebo group. Similar results have been obtained by Cremonini et al. (2002) and Duman et al. (2005).

##### Clostridium difficile associated diarrhea

The effects of *S. boulardii* on *C. difficile* have also been studied. *C. difficile* is responsible for 20% of antibiotic associated diarrhea cases (Kelly et al., 1994; Krämer and Bischoff, 2006) and causes pseudomembranous colitis, an infection of the colon. There are only two standard antibiotics for *C. difficile* infection, namely vancomycin and metronidazole, and the response rate to the later has been declining (McFarland, 2007). *S. boulardii* in combination with antibiotics has been shown effective for treating *C. difficile* associated diarrhea and colitis (Mcfarland et al., 1994; McFarland, 2010; Surawicz et al., 2000). In a randomized placebo-controlled trial of 124 patients suffering from *C. difficile* infection (Mcfarland et al., 1994), standard antibiotics were combined with *S. boulardii* (500 mg/day) or placebo. Patient follow-up revealed a significant reduction in *C. difficile* infection reoccurrence in those treated with *S. boulardii* (34.6% compared to 64.7% for the placebo, *P* = 0.04). In a similar study involving 170 patients (Surawicz et al., 2000), reoccurrence in the *S. boulardii*-treated group was 16.7% compared to 50% for the placebo group (*P* = 0.05). In a more recent comparison of the efficacy of a mixture of probiotics, McFarland et al. (2006) concluded that only *S. boulardii* was effective against *C. difficile* infection.

The effects of *S. boulardii* have also been studied *in vitro* and in animal models such as gnotobiotic mice (Elmer and Corthier, 1991; Elmer et al., 1999; Castex et al., 1990) rats (Karen et al., 2010; Sezer et al., 2009), and turkeys (Bradley et al., 1994). Czerucka and Rampal (2002) linked the effects of *S. boulardii* to the production of a 54-KDa protease. Its mechanism appears to degrade *C. difficile* toxins (Castagliuolo et al., 1996; Qamar et al., 2001), and could do likewise to the colonic cell surface receptors for *C. difficile* (Pothoulakis et al., 1993; Castagliuolo et al., 1996; Pothoulakis, 2009; Castagliuolo et al., 1999), or otherwise inhibit *C. difficile* attachment to intestinal cells (Buts and Bernasconi, 2005; Buts, 2009; Tasteyre et al., 2002). *S. boulardii* also appears to increase the immune response to *C. difficile* toxins A and B (Buts, 2009). It has been demonstrated that *S. boulardii* stimulates an increase in intestinal immunoglobin A secretion during a *C. difficile* toxin A challenge in mice (Qamar et al., 2001; Figure 1).

##### Traveler’s diarrhea

Each year million of people worldwide develop traveler’s diarrhea. *S. boulardii* activities in the prevention of traveler’s diarrhea have been widely investigated. In 1016 travelers visiting various countries in the world Kollaritsch et al. (1993) reported a significant reduction in diarrhea among patients receiving 5 billion CFU of *S. boulardii*/day (34% versus 40% in patients receiving placebo *P* = 0.019). Based on analysis of 12 studies of the use of probiotics to prevent traveler’s diarrhea, McFarland (2007) reported a significant benefit of two probiotics, namely *S. boulardii* and a mixture of *Lactobacillus acidophilus* and *Bifidobacterium bifidum*.

##### Acute diarrhea in adults and children

Every year, an estimated 2 million deaths worldwide occur as a result of acute diarrhea (Billoo et al., 2006). Several studies have shown the beneficial effects of *S. boulardii* in preventing acute diarrhea. In a group of 50 children (aged 2 months to 2 years) receiving 10 billion CFU of *S. boulardii*/day or a placebo in combination with oral rehydration salt and nutritional support, Billoo et al. (2006) showed significant reductions in stool frequency and duration of diarrhea in the *S. boulardii* group compared to the placebo group. In a double-blind randomized study, Kurugöl and Koturoglu (2005) noted a significant reduction in the number of days with diarrhea and hospitalization among 200 children treated with *S. boulardii*. Similar results were found in another double-blind randomized study by Villarruel et al. (2007).

##### Tube-feeding-associated diarrhea

DeMeo et al. (1998) estimated that approximately 68% of tube-fed patients develop diarrhea. Several studies suggest the beneficial effects of *S. boulardii* in restoring normal intestinal microflora and preventing tube-feeding-associated diarrhea. In a double-blind placebo-controlled trial involving 40 tube-fed patients, a 50% reduction in days with diarrhea was observed among patients given *S. boulardii* compared to the placebo group (Tempé et al., 1983). Another double-blind placebo-controlled study following 128 critically ill tube-fed patients (Bleichner et al., 1997) showed a significant but small reduction in days with diarrhea among patients given 40 billion CFU of *S. boulardii* four times/day (14.2 versus 18.9% in patients receiving placebo).

##### Inflammatory bowel disorders

Inflammatory bowel diseases (IBD), Crohn’s disease, ulcerative colitis and Irritable bowel syndrome are chronic inflammatory disorders of the gastrointestinal tract. Numerous studies showed that *S. boulardii* hold promise for the treatment of inflammatory bowel disorders. In a double-blind study of 20 patients suffering from Crohn’s disease (Plein and Hotz, 1993) showed a significant reduction in the bowel movement among patients receiving *S. boulardii* in addition to their conventional therapy. In a single-blind study of 32 patients with Crohn’s disease (Guslandi et al., 2000), similar results were reported for patients receiving *S. boulardii* (20 × 10^9^ CFU/day) compared to patients receiving 500 mg of mesalazine three times daily. Guslandi et al. (2003) reported improvement of 68% of patients with ulcerative colitis receiving mesalazine (3 g/day) and 250 mg of *S. boulardii* in capsules three times daily for 4 weeks. Finally, *S. boulardii* has been shown to exert promising beneficial effect in the treatment of IBD. One suggested mechanism unique action on inflammation by specific alteration of the migratory behavior of T cells, which accumulate in mesenteric lymph nodes (Dalmasso et al., 2006a,b). *S. boulardii* treatment might thus limit the infiltration of T-helper 1 cells, and hence colonic inflammation and amplification thereof by pro-inflammatory cytokines.

Further *in vivo* studies are required to investigate the ability of various yeasts strain to exercise their probiotic effect (Foligné et al., 2010). Also, further studies are requiring an optimal and controlled probiotic formula to enhance the biotherapeutic effects of yeast.

##### Chronic diarrhea in human immunodeficiency virus and others

In a randomized double-blind study of 35 patients with AIDS-related diarrhea, Saint-Marc et al. (1995) reported a reduction in diarrhea among patients receiving *S. boulardii* (3 g/day for 7 days). After 1 week of treatment with *S. boulardii*, 61% of the patients were diarrhea-free compared with 12% in the placebo group. Finally, other studies revealed the efficacy of *S. boulardii* to reduce diarrhea in people suffering from giardiasis (Besirbellioglu et al., 2006), amebiasis (Mansour-Ghanaei et al., 2003; Tanyuksel and Petri, 2003) where *S. boulardii* reduces the number of red cells adhering to amoeba and the number of amoebae bearing red cells (Rigothier et al., 1994), and *Helicobacter pylori* gastritis (Duman et al., 2005). It was reported that *S. boulardii* had improved the post-treatment dyspepsia symptoms of *H. pyloris* infection without having a significant effect on the rate of *H. pylori* eradication (Cindoruk et al., 2007). Recently, Vandenplas et al. (2009) suggested that *S. boulardii* alters the structure of *H. pylori*. Further studies are required for better understanding of yeast probiotics mechanisms.

##### Probiotic bacteria/yeast interaction

Because probiotic yeast and bacteria have different mechanisms of action, a synergetic effect and higher viability might be expected from mixing both types of probiotics (Bisson et al., 2010 and Suharja et al., 2012). Several studies showed that yeasts could positively interact with probiotic bacterial by enhancing their survival and stimulating their growth (Gobbetti et al., 1994; Liu and Tsao, 2009a,b,c; Katakura et al., 2010; Suharja et al., 2012). This positive interaction between yeast and bacteria might be attributed to the production of nutrients such as peptides, amino acids, and/or vitamins (Gobbetti et al., 1994; Viljoen, 2001, 2006; Narvhus and Gadaga, 2003; Kawarai et al., 2007; Katakura et al., 2010). It is well known that the cell wall of yeasts is mainly composed of glucans, mannans, and chitin, all of which may play a role in co-aggregation and cohesion phenomena which play a major role in the survival of probiotic bacteria (Chaffin et al., 1998; Millsap et al., 1998). Aggregation may involve the yeast mannans form a capsule-like structure where bacteria may associate with sugars by means of a lectin-like (Millsap et al., 1998). Katakura et al. (2010) identified proteins on the surface of *Lactococcus lactis* IL1403, which recognize the yeast mannan involved in adhesion of lactic acid bacterium to the yeast. Also, in the study of interactions between microorganisms present in kefir grains, Golowczyc et al. (2009) showed that thermolabile non-covalently lectin-like surface proteins of several *Lactobacillus* kefir strains can mediate the aggregation with *S. liplytica* CIDCA 812 yeast cells. It has been hypothesized by Xie et al. (2012) that the aggregation of LAB with yeasts in gastric or intestinal juices might have positive effects on enhancing the tolerance of LAB. The same group concluded that proteins of the cell surface of *Labacillus paracasei* H9 and polysaccharides in cell walls of *S. cerevisiae* play important roles in co-aggregation of the two strains and the microbial adhesion specificity to Caco-2 cells that contributes to enhancing probiotic potentials of *L. paracasei* H9. Also, Liu and Tsao (2010a,b) reported that the use of *Williopsis saturnus* var. *saturnus* enhances the survival of the two probiotic bacteria *L. bulgaricus* and *L. rhamnosus* in fermented milk. The same group concluded that the use of yeast enhance the stability of probiotic bacteria in fermented milk thereby extend the product shelf-life and retain nutritional value (Liu and Tsao, 2010a,b).

#### Other medical application

The relatively recent discovery of bio-drug effects of yeasts potentially opens a door to new clinical applications. Blanquet et al. (2001) defined bio-drugs as orally administered living recombinant microorganisms that express disease-fighting proteins. The application of non-recombinant yeast as antimycotics for therapeutic treatment of human and animal fungal infection has also received considerable attention (Polonelli et al., 1986; Hodgson et al., 1995; Séguy et al., 1998; Conti et al., 2002; Schmitt and Breinig, 2002). The killer toxin of *L. mrakii* has been proposed as an antifungal compound against *Candida* spp., due to its similarity to aculeacin and stability to pH and temperature changes (Walker et al., 1995; Hodgson et al., 1995). Weiler and Schmitt (2003) found that the zygocin, produced by *Zygosaccharomyces bailii*, has a rapid killing effect against a wide range of pathogenic yeasts including *Candida albicans*, *Candida glabrata* and *Candida krusei*, and *Sporothrix schenckii* based on disruption of membrane ion gradients. However, it is important to note that killer toxins are large glycoprotein compounds and hence capable of inducing an immune response in the host. To deal with this potential challenge, several strategies have been proposed. For example, Magliani et al. (1997) synthesized small non-antigenic peptides with killer activity. The same group suggested the utilization of an anti-idiotypic antibodies from killer toxin secreted by *W. anomalus* (Magliani et al., 2008). These “antibiobodies” (i.e., immunoglobulin molecules acting directly to provide passive immunity without involvement of other immune system factors) showed a significant microbicidal activity against wide range of pathogenic agents through the interaction with specific killer toxin receptors composed by beta-glucans. A novel antifungal vaccine derivative of *W. anomalus* killer toxin (PaKT) has been described recently by Polonelli et al. (2011).

### Veterinary applications

Yeast as a source of protein, vitamins, and minerals in animal feeds and for veterinary use has a long history (Anupama and Ravindra, 2000; Bekatorou et al., 2006). Yeast single cell protein is effective for accelerating growth and improving the well-being of cattle by stimulating rumen acetogens (Halász and Lásztity, 1991; Klein and Favreau, 1995; Kurtzman et al., 2011a,b). The use of yeast cell wall polysaccharides as adjuncts for animal and fish feeds has been found to improve the health of growing pigs (Sauerwein et al., 2007). Increasing resistance of bacterial pathogens to antibiotics, due largely to overuse of these compounds in livestock production, has fueled the search for alternative strategies such as probiotics to ensure animal health (Dawson et al., 1990; Li et al., 2006; van Heugten et al., 2003). *S*. *cerevisiae* was has been used in many probiotic preparations (Lyons et al., 1993; Edens, 2003; Swanson et al., 2002a,b). Yeast may act by various mechanisms such as modulation of immune system, histamine release (Holck et al., 2007), binding to toxins, and to pathogenic cell (Castex et al., 1990; Elmer and Corthier, 1991; Elmer and McFarland, 1987), interactions with gut constituents, and anti-tumor activities (Ghoneum et al., 2007).

Several studies of poultry have shown that *S*. *boulardii* increases bird weight when used as a feed supplement during the early stages of life (Bradley et al., 1994; Hooge, 2004; Gao et al., 2008; Patterson and Burkholder, 2003). Investigating the effect of 1–100 g of dried *S. boulardii*/kg of feed, Line et al. (1998) found that colonization of the cecum of broiler chickens challenged with *Salmonella typhimurium* (3.2 × 10^8^ CFU) was 5–20% and inversely proportional to the amount of yeast given, compared to 70% in birds not fed the yeast. Investigating the impact of viable cells (6.5 × 10^10^ CFU/kg of feed), extract, or cell wall of *S. cerevisiae* on growth, meat quality, and ileal mucosal development in male broiler chickens, Zhang et al. (2005) concluded that whole yeast improved growth rate as well as meat tenderness and oxidative stability, while cell wall improved ileal mucosal development only. Other authors have obtained similar results with broiler chickens and other animals (Lammerding et al., 1988; Morales-López et al., 2010).

Positive results have also been obtained in ruminants. Until recently, the most consistent beneficial effect of feeding *S. cerevisiae* to ruminant has been stabilization of the rumen pH or prevention of acidosis caused by rapid fermentation of large quantities of carbohydrates (Chiquette, 2009). It has been demonstrated *in vitro* that *S. cerevisiae* can stimulate the digestion of cellulose by cellulolytics organisms such as *Fibrobacter succinogenes* and *Ruminococcus flavefaciens* and promotes the utilization of lactate by *Megasphaera elsdenii* and *Selenomonas ruminantium* (Callaway and Martin, 1997). It is also well known that feeding yeast stimulates rumen microbial growth and oxygen scavenging, thus creating more favorable conditions for anaerobic microorganisms (Fonty and Chaucheyras-Durand, 2006). Finally, the mode of action of probiotic yeast in the rumen depends on several factors such as yeast strain, viability, and the composition of the diet of the animal (Chiquette, 2009).

The applications of yeasts in human foods and animal feeds as well as in agriculture and other sectors are increasing and market demand is providing motivation to continue or even increase research and development in this field. Only future studies will reveal the ultimate potential of these microorganisms in different fields of application.

## Conflict of Interest Statement

The authors declare that the research was conducted in the absence of any commercial or financial relationships that could be construed as a potential conflict of interest.
